# Validation study on light scattering changes in kiwifruit during postharvest storage using time-resolved transmittance spectroscopy

**DOI:** 10.1038/s41598-023-43777-5

**Published:** 2023-10-02

**Authors:** Te Ma, Tetsuya Inagaki, Satoru Tsuchikawa

**Affiliations:** Graduate School of Bioagricultural Sciences, Nagoya University, Furo-cho, Chikusa, Nagoya, 464-8601 Japan

**Keywords:** Applied optics, Plant physiology

## Abstract

Visible and near-infrared spectroscopy has been well studied for characterizing the organic compounds in fruit and vegetables from pre-harvest to late harvest. However, due to the challenge of decoupling of optical properties, the relationship between the collected samples’ spectral data and their properties, especially their mechanical properties (e.g., firmness, hardness, and resilience) is hard to understand. This study developed a time-resolved transmittance spectroscopic method to validate the light scattering changing characteristics in kiwifruit during shelf-life and in cold storage conditions. The experimental results demonstrated that the reduced scattering coefficient (${\mu }_{s}^{\prime}$) of 846 nm inside kiwifruit decreased steadily during postharvest storage and is more evident under shelf-life than in cold storage conditions. Moreover, the correlation between the ${\mu }_{s}^{\prime}$ and the storage time was confirmed to be much higher than that using the external color indexes measured using a conventional colorimeter. Furthermore, employing time-resolved profiles at this single wavelength, an efficacious mathematical model has been successfully formulated to classify the stages of kiwifruit softening, specifically early, mid-, and late stages. Notably, classification accuracies of 84% and 78% were achieved for the shelf-life and cold storage conditions, respectively.

## Introduction

Kiwifruit is a typical climacteric fruit; the ripening-associated events continue to develop after being harvested^1,2^. Since excessive fruit ripening during storage leads to postharvest losses, it is generally harvested pre-climacteric to ensure its most extended storage^3^. However, the picked fruit generally vary in their degree of ripeness, and there are variations in the degree of climacteric ripening (e.g., color change, sugar accumulation, and decay of firmness) during the transport and storage periods.

Most commercial quality classification systems rely on the external appearance of agricultural products (e.g., color, size, and surface damage). Typically, colorimeters in the fruit industry express color using L^*^, a^*^, and b^*^ values. The L^*^ represents brightness, the a^*^ value ranges from green to red, and the b^*^ value ranges from blue to yellow^4^. Other color indexes (e.g., a^*^/b^*^, Chroma, Hue) have also been used as valuable indicators of fruit ripeness^5,6^. However, in contrast to other fruits, kiwifruit exhibits minimal visible changes in skin color during ripening^7^. The evaluation of taste and texture parameters often relies on several widely employed methods, including assessing color changes through kiwifruit cutting^8^, conducting Magness-Taylor (MT) firmness testing^9^, and employing Brix refractometry^10^. However, these conventional approaches suffer from limitations such as being time-consuming and destructive. Consequently, it is advisable to pursue the development of non-destructive, convenient, and resilient evaluation methods to maintain postharvest quality, reduce waste, and promote sales.

Visible and near-infrared (Vis–NIR) spectroscopy has been studied and developed in fruit and vegetable quality assessments, such as in the evaluation of acidity and soluble solids content (SSC)^11–13^. Generally, the absorption coefficient designated as *μ*_a_ and the reduced scattering coefficient designated as ${\mu }_{s}^{\prime}$ are used to characterize light absorption and light scattering process in highly scattering media. Specifically, the ${\mu }_{s}^{\prime}$ can be defined as ${\mu }_{s}^{\prime}= (1- g)\, {\mu }_{\mathrm{s}},$ where *g* represents the anisotropy factor governing the direction of light, and ${\mu }_{s}$ represents the scattering coefficient^14^. It is well known that the spectral intensities collected by conventional spectrometry are affected by the samples’ varying physical structures^15^. However, since the decoupling of optical properties continues to pose significant challenges^16^, most conventional studies use additional spectral pre-treatments to determine the chemical compounds^17,18^. Additionally, chemometrics has been used to calibrate the spectral data with the reference data obtained through traditional standard methods^18,19^. However, these conventional approaches present significant challenges in understanding the relationship between spectral data from collected samples and their mechanical properties, and detecting chemical changes associated with the firmness of fruit remains a substantial challenge^13,20–22^. Furthermore, it is difficult to develop a fruit quality evaluation system based on a single or multiple wavelengths.

Considerable efforts have been made toward using spatially resolved spectroscopy (SRS) method to evaluate the optical properties by measuring the reflectivity at different distances from a steady-state spotlight source^23–26^. In contrast, time-resolved spectroscopy (TRS) method uses pico- or femtosecond laser pulses, sensitive photodetectors, and fast-timing acquisition electronics. The multiply scattered and attenuated diffusely reflected or transmitted pulses are detected and quantified using theoretical approaches to study the light absorption and scattering characteristics. The use of the TRS for the optical characterization of highly scattering media has been introduced in the biomedical field in the early 1990s^27^. In the early 2000s, the application to the food sector was pioneered by Cubeddu et al.^28^ and Tsuchikawa^29^. Since the TRS is considered as a more accurate method for measuring optical properties^30^, it has been used to validate light scattering information in developing a portable SRS-based fruit quality evaluation system^31^. Indeed, time-resolved reflectance spectroscopy (TRRS) has been mainly used to study optical property changes in fruit. Zerbini et al.^32^ used the TRRS to detect brown heart in pears. Valero et al.^33^ used the TRRS to separate the scattering effects from that of light absorption in kiwifruit and correctly classify the used samples into three groups, achieving performance results of 75% for firmness, 60% for sugar content, and 97% for acidity. Zerbini et al.^34^ measured the optical characterizations in nectarines at 600–1100 nm using the TRRS. They discovered that the ${\mu }_{\mathrm{a}}$ at 670 nm (i.e., the absorption of chlorophyll) was well related to the maturity. Van Beers et al.^35^ reported that during the ‘Braeburn’ apple’s maturation, light scattering decreased in the apple cortex, whereas the skin scattering increased in the two bicolored cultivars. Additionally, the ${\mu }_{\mathrm{a}}$ could explain 13–34% of the firmness variations. In comparison to TRRS, time-resolved transmittance spectroscopy (TRTS) offers several advantages. Theoretically, TRTS allows for effective correction of optical transmission depth fluctuations based on sample diameter or thickness, and is less susceptible on the impact of sample surface curvatures^31,36^. It is well known that the typical visible-NIR absorption spectrum of fruit is dominated by the water peak near 970 nm and the chlorophyll peak near 675 nm, whereas the wavelengths at approximately 800–900 nm have extremely low light absorption^35,37^. Such specific wavelengths are more suitable for studying light scattering characteristics in fruit, although the spectral absorbance information is deficient for chemical composition analysis.

This study aimed to evaluate kiwifruit’s light scattering changing characteristics, mainly caused by mechanical property changes during postharvest storage. The objectives of this study are summarized as follows: (1) develop a TRTS measurement system that mainly includes a picosecond pulsed laser with a wavelength of 846 nm and a streak camera; (2) validate ${\mu }_{s}^{\prime}$ changing characteristics in kiwifruit during shelf-life and in cold storage conditions; (3) benchmark the performance on monitoring the ripening and postharvest decay using the ${\mu }_{s}^{\prime}$ against conventional color indexes; and (4) develop a new nondestructive method to classify early, mid-, and late kiwifruit softening using the time-resolved profiles of 846 nm.

## Materials and methods

### Sample preparation

In this study, ‘Hayward’ kiwifruit (*Actinidia chinensis* var. *deliciosa*) under pre-climacteric conditions were commercially purchased from a local fruit market in the Wakayama Prefecture of Japan. and such kiwifruit are provided around February each year for fruit ripening experience at home. The skin of this species is thin but has hair and is irregular, which makes it challenging to use conventional reflection spectroscopic methods for quality evaluation.

### TRTS measurement system

**Figure 1:**
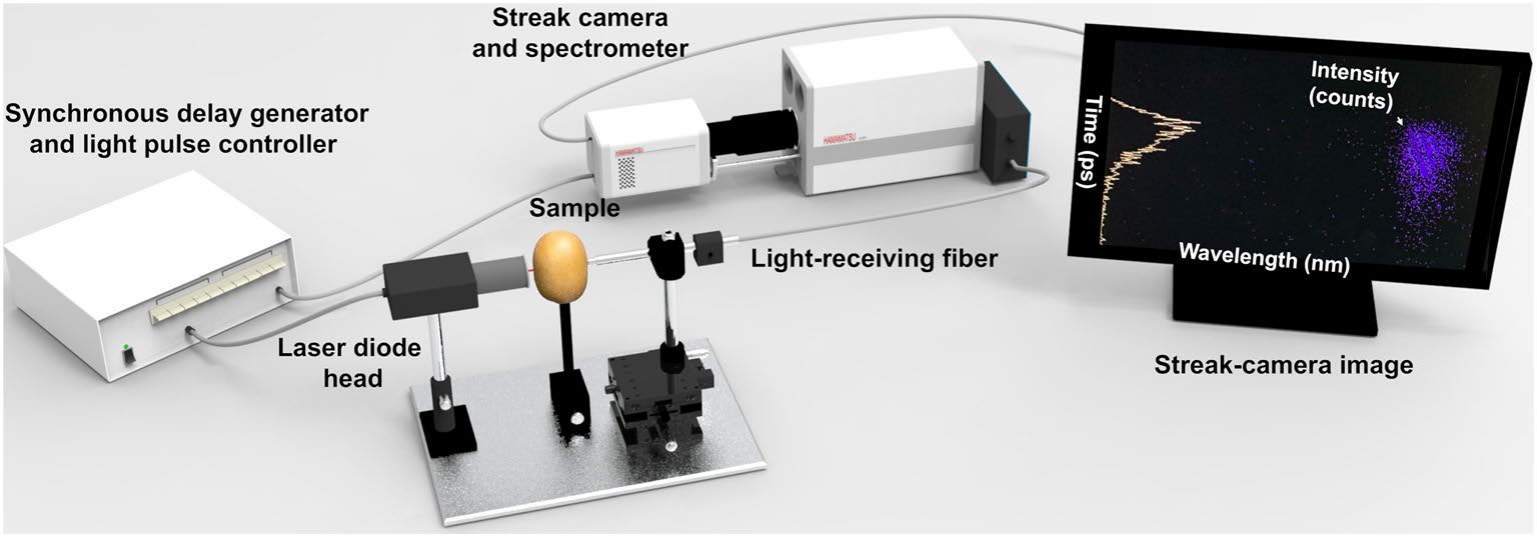
Main components of the developed TRTS measurement system.

Figure 1 shows the three main components of the TRTS system, which include an 846 nm picosecond pulsed laser at 70 ps pulse width and 151 mW peak power (PLP-10; Hamamatsu Photonics Co., Hamamatsu, Japan), a synchronous delay generator, and a streak camera with a time resolution of 10.3 ps (C5680; Hamamatsu Photonics Co., Hamamatsu, Japan). First, a circular laser beam on the sample of approximately 1.5 mm in diameter was adjusted. Then, the transmitted photons exiting the sample were collected using a step-index fiber with a diameter of 300 μm and a numerical aperture of 0.22 ± 0.02 (A5760-02; Hamamatsu Photonics Co., Hamamatsu, Japan), placed in contact with the sample. Finally, the time variation of the transmitted radiation intensity was recorded using the streak camera. It can register the time signal of the transmitted light through a photo-counting sensor and a time-acquisition board. The instrument response function (IRF) was measured using a couple of neutral density filters with 1% transmission ratio. The samples’ TRP was acquired along the equator line through the sample’s skin using a time range of 5 ns, and photon counting was performed for 60 s. Measurements on aqueous solutions of intralipid were done to provide evidence of the performance of the developed TRTS system in recent work^31^.

### Check the 846-nm light absorption and scattering characteristics of sliced kiwifruit samples

Three samples at almost the same commercial maturity stage were first measured using the designed TRTS system by slicing them. Their cross-sectional microscopic images were then taken using a bright-field microscope (PrimoStar, Carl Zeiss Microscopy Co., Japan).

### Check the 846-nm light absorption and scattering characteristics of whole kiwifruit samples under cold storage condition

A total of 80 samples derived from the same batch, were subjected to non-destructive measurements utilizing the identical TRTS system for 7-day intervals from 0 day (i.e., after arriving at the laboratory) to 42 days under cold condition (4 °C ± 2 °C with 75% RH), until one sample experienced severe damage.

### Evaluate the efficacy of the ${\mu }_{s}^{\prime}$ in monitoring ripening in comparison with conventional color indexes

A total of 110 samples belong to the same batch were prepared. The firmness, SSC, and pH of 10 samples were measured immediately after arriving at the laboratory (as 0 day). To compare the quality changes under different postharvest storage conditions, 50 kiwifruit samples were randomly selected and stored under shelf-life (22 °C ± 2 °C with 65% RH) condition. Their time-resolved pulse (TRP) spectra were non-destructively measured by the developed TRTS system. Then, each ten individual kiwifruits were randomly picked at 2-day intervals from 1 day, the TRP spectra, firmness, SSC, and pH were measured on each occasion until 7 days. To estimate the light scattering changes at 846 nm during postharvest storage, the last ten samples were non-destructively measured by the developed TRTS system on each occasion until 13 days, when one of the samples had visually rotted. Finally, their firmness, SSC, and pH values were measured.

Meanwhile, 50 remained samples were stored under the same cold (4 °C ± 2 °C with 75% RH) condition. As the same data measurement steps as shelf-life condition, their TRP spectra were non-destructively measured by the developed TRTS system. Then, every ten samples were randomly picked at 7-day intervals from 7 days. Their TRP spectra, firmness, SSC, and pH were measured for up to 1 month. In comparison, the last ten samples were non-destructively measured using the same TRTS system until 49 days, when one was severely damaged. Finally, their firmness, SSC, and pH values were measured. Notably, to reduce the temperature-induced spectral variations, the samples were stored at room temperature conditions for approximately 6 h before conducting the measurements. Other pre-treatments (e.g., the use of 1-methylcyclopropene) were not used.

### Non-destructive measurement of external color and diameter

A portable colorimeter (SC-10 precision colorimeter, 3nh Technology Co., Ltd., Shenzhen, China) was employed to ascertain the skin color of the kiwifruit's equatorial region after each TRP measurement. The possibilities for expressing the color were selected as L^*^, a^*^, b^*^, a^*/^b^*^, *Chroma* = $\sqrt{{a}^{*2}+{b}^{*2}}$, or *Hue* = tan^−1^ (b^*/^a^*^). Subsequently, the sample diameter was measured using a digital caliper (0.01 mm accuracy) to correct the optical path effects in the TRP.

### Destructive measurement of firmness, SSC, and pH reference values

Following various postharvest storage durations, MT firmness assessments were administered on the identical equatorial region previously employed for the measurement of TRP. The sample’s skin was first removed, and the pulp was destructively measured using a 5-mm-diameter plunger (Fudoh Rheo Meter, Rheotech, Inc., Tokyo, Japan) at a speed of 1 mm/s. Then, the fresh juice of the same equatorial area was extracted for traditional SSC measurement using a Brix refractometer (IPR-201, Spittz, Atago Co., Ltd., Tokyo, Japan), pH measurements were recorded using a pH meter (LAQUAtwin-pH-22B, Horiba Advanced Techno Co., Ltd., Kyoto, Japan).

### Time-resolved profile analysis

The measured raw TRP spectral data were first smoothed by a Savitzky–Golay finite impulse response smoothing filter (polynomial order, 2; frame length, 5). Principal component analysis (PCA) was then used to extract the characteristic changes in the TRP spectra. The PCA is a non-parametric, unsupervised tool for reducing the dimensionality of spectral data, increasing interpretability while minimizing information loss simultaneously^38–40^. The PC loadings were used to generate the PC scores, which were then utilized to validate the correlation between the storage time of kiwifruit (i.e., the softening process) and the estimated ${\mu }_{s}^{\prime}$ using the curve-fitting method. The values of ${\mu }_{a}$ and ${\mu }_{s}^{\prime}$ were computed by fitting the convolution between the IRF and the model solution^41^. Theoretically, the number of photons arriving at the detector per unit area per unit time can be expressed as follows:

1 $$T\left(\rho,t\right)=-{\mathrm{exp}}\left({-\mu }_{a}vt-{\rho }^{2}/4Dvt\right)/{2(4\pi Dv)}^{3/2}{t}^{5/2}\sum_{m=-\infty }^{+\infty }[{\mathcal{Z}}_{1,m}{\mathrm{exp}}\left(-{{\mathcal{Z}}_{1,m}}^{2}/4Dvt\right)-{\mathcal{Z}}_{2,m}{\mathrm{exp}}\left(-{{\mathcal{Z}}_{2,m}}^{2}/4Dvt\right)]$$

2 $${\mathcal{Z}}_{1,m}=s\left(1-2m\right)-4m{\mathcal{Z}}_{e}-{\mathcal{Z}}_{0}$$

3 $${\mathcal{Z}}_{2,m}=s\left(1-2m\right)-\left(4m-2\right){\mathcal{Z}}_{e}+{\mathcal{Z}}_{0}$$

4 $${\mathcal{Z}}_{0}=1/{\mu }_{s}^{\prime}$$

5 $${\mathcal{Z}}_{e}=2AD$$

6 $$D=1/3{\mu }_{s}^{\prime}$$

where $\uprho$ is the distance from the light incident center, which was set as 0 for the transmittance measurement in this study; *t* is time; *s* is sample thickness; *v* is the speed of light in a vacuum dividing by the sample's index of refraction (*n*); A and n were set as 2.58 and 1.34, respectively; seven dipoles (m = 0, ± 1, ± 2, ± 3) were used. In this study, the entire TRP was used for fitting. The fitting range for ${\mu }_{s}^{\prime}$ was set from 0.1 to 10 mm^−1^; and from 0.0001 to 0.1 mm^−1^ for ${\mu }_{a}$, because the ${\mu }_{s}^{\prime}$ was significantly larger than ${\mu }_{a}$ at the 846 nm light wavelength. The fitting method was trust-region-reflective.

### Develop a mathematical model to classify early, mid-, and late kiwifruit softening using the single 846-nm TRP spectra

To achieve the initial value (i.e., kiwifruit initial quality variation) correction purpose, the TRP difference spectra were obtained by subtracting the TRP spectra collected at 0 day from the spectra that were measured with the firmness measurements. PCA was then used to extract the characteristic changes in the TRP difference spectra. Early, mid-, and late kiwifruit softening stages were classified based on the measured firmness values. The representative PC scores were further used to classify the three softening stages that under shelf-life and cold storage conditions, respectively, via the support vector machine (SVM) method^42^. Five-fold cross-validation was used to against overfitting. This study used Matlab (The MathWorks Inc., Natick, MA, USA) for data analysis and image processing.

## Results

### 846-nm light absorption and scattering characteristics of sliced kiwifruit samples

**Figure 2:**
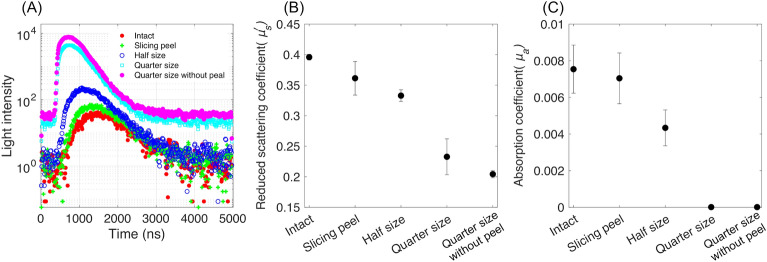
Optical properties collected from kiwifruit samples by cutting them (**A**) TRP, (**B**) ${\mu }_{s}^{\prime}$, and (**C**) *μ*_a_.

**Figure 3:**
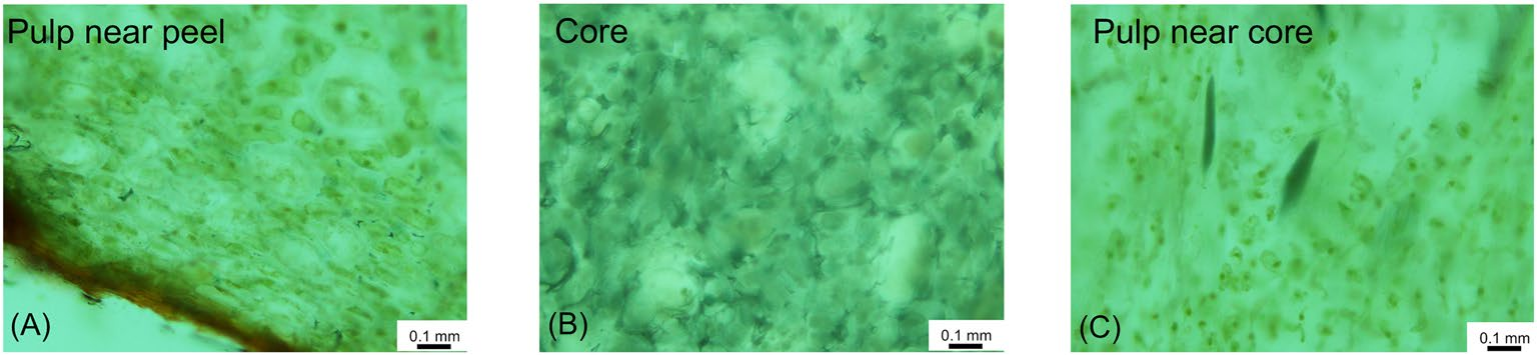
Microscopic images of different parts of the same kiwifruit.

Figure 2A shows the TRP collected by cutting the same kiwifruit sample. The intact fruit has the lowest light intensity, and the quarter size without peel (i.e., the pulp) has the highest value. Figure 2B,C show the estimated ${\mu }_{s}^{\prime}$ and *μ*_a_ of the total three samples by fitting their TRP. Their firmness values were 23.52, 22.05, and 21.08 N/cm^2^, and their diameters were 51.7, 51.8, and 50.2 mm. Overall, the estimated *μ*_a_ was much lower than the ${\mu }_{s}^{\prime}$, indicating that the light at 846 nm could penetrate and considerably scatter in the whole kiwifruit. The ${\mu }_{s}^{\prime}$ difference between the half and quarter sizes was relatively significant, suggesting that light scattering in the core parts was stronger than in the flesh part. This could be because the central core parts are fibrous (Fig. 3), and a high difference in the refractive index between the core and the flesh.

**Figure 4:**
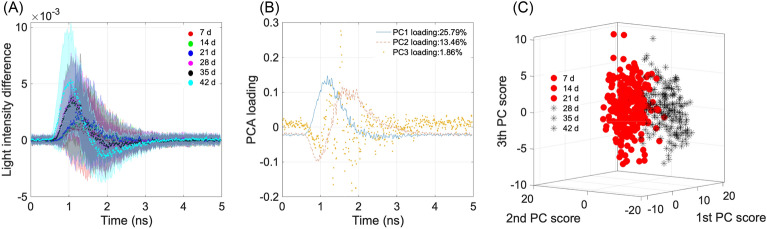
(**A**) TRP difference spectra of the 80 samples under cold storage, (**B**) the first three PC loadings, (**C**) scatter plot of the first three PC scores.

The TRP difference spectra of the 80 samples subjected cold storage are depicted in Fig. 4A. It is apparent from the spectra that the transmitted light exhibited an increase in intensity (up-shift) and a decrease in temporal duration (left-shift) as the storage time progressed. Previous studies have indicated some characteristic changes during the fruit-ripening process, including cell wall depolymerization and water-soluble pectin increases^43–47^. Furthermore, Newman and Redgwell^48^ used nuclear spin relaxation experiments to show that kiwifruit’s firmness was proportional to the content of rigid non-cellulosic matter in the cell wall. These factors could lead to an overall trend decrease in light scattering despite the expected increase in light scattering caused by water loss. Figure 4B displays the first three PC loadings, which represent the weights assigned to each variance value for calculating principal component (PC) scores. The cumulative contribution rate of the first three PCs amounted to 41.11%. Notably, a strong correlation was found between PC1 loading and the intensity of transmitted light. Additionally, PC2 loading exhibited relatively high absolute values at approximately 1 and 1.5 ns, suggesting a potential influence from the speed of transmitted light. In contrast, the cumulative contribution rate of PC3 loading was extremely low, underscoring its utility in capturing variance from the original input spectra. Figure 4C shows the scatter plot of the first three PC scores, effectively demonstrating the significant changes observed in the TRP difference spectra after a 3-week period of cold storage.

### Statistics of external color, weight loss, and diameter during postharvest storage

**Figure 5:**
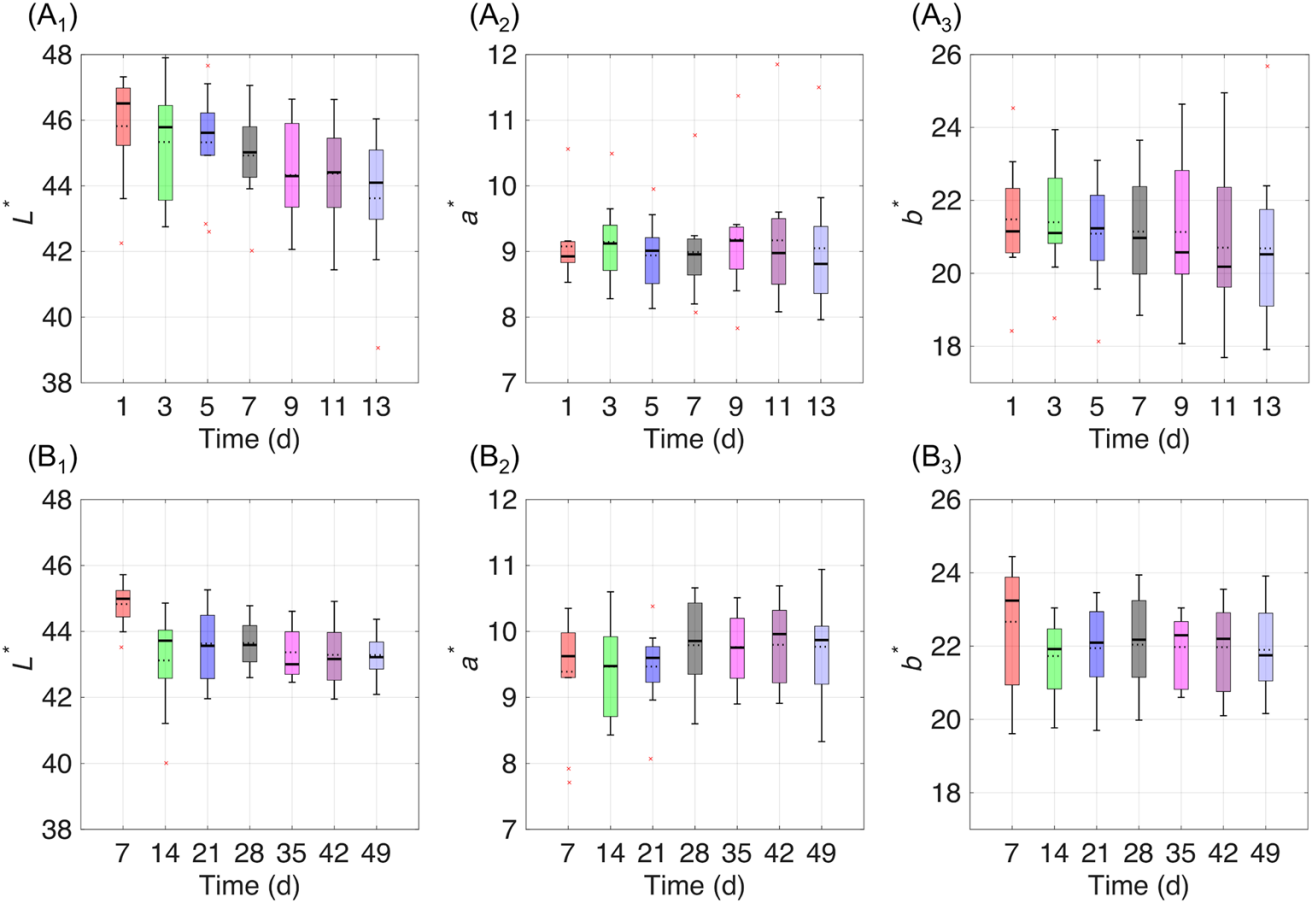
Outside color change of the ten kiwifruits during (**A**) shelf-life and in (**B**) cold storage conditions, respectively.

Figure 5 shows the boxplot of the outside color change of the ten kiwifruits during (A) shelf-life and in (B) cold storage conditions, respectively. The whiskers indicate the most extreme data points not considered outliers that are plotted individually using the ‘×’ marker symbol. Tables 3 and 4 summarize the correlation coefficients (*r*) between the color parameters and the storage time. Previous studies^7,8^ indicated that under storage time, the L^*^ value of fresh-cut kiwifruit slices tended to decrease with an increase in a^*^ values. This study demonstrates that the color changes on the kiwifruit skin were not noticeable enough, showing the correlation to storage time with large fluctuations. It suggests the limitations of using conventional color-based methods to monitor the postharvest decay of kiwifruit non-destructively.

### Statistics of firmness, SSC, and pH reference values

**Figure 6:**
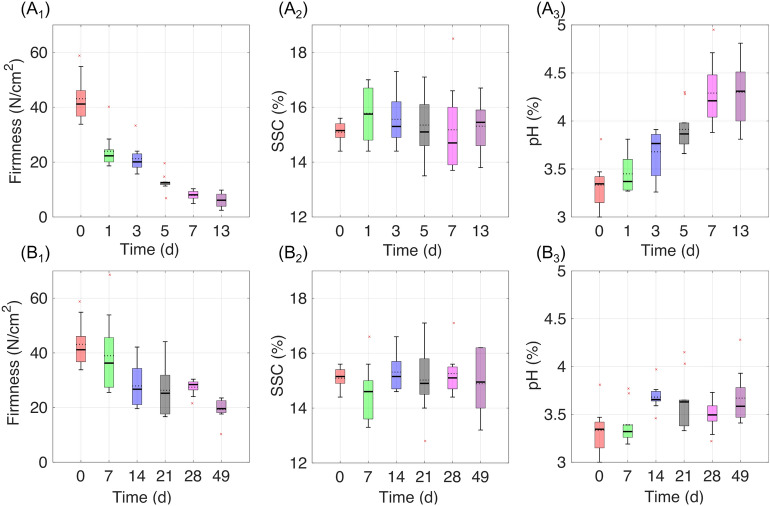
Changing tendency of firmness, SSC, and pH under (**A**) shelf-life and (**B**) cold storage conditions, respectively.

Overall, the time and temperature of storage fundamentally influence the diminution of firmness and pH of the kiwifruit samples. Figure 6 shows the changing tendency of firmness, SSC, and pH of the kiwifruit samples under (A) shelf-life and (B) cold storage conditions, respectively. The rates of the decrease in firmness and the increase in pH were different at low temperatures. In contrast, the SSC was less changing, which is not in agreement with most previous studies, in which SSC increased with storage time^49,50^. This could be because the purchased kiwifruit samples initially had variability in the degree of SSC values, and their values did not change as significantly as the other two properties during the storage.

### Investigation of 846-nm light absorption and scattering characteristics of intact kiwifruit samples during postharvest storage

**Figure 7:**
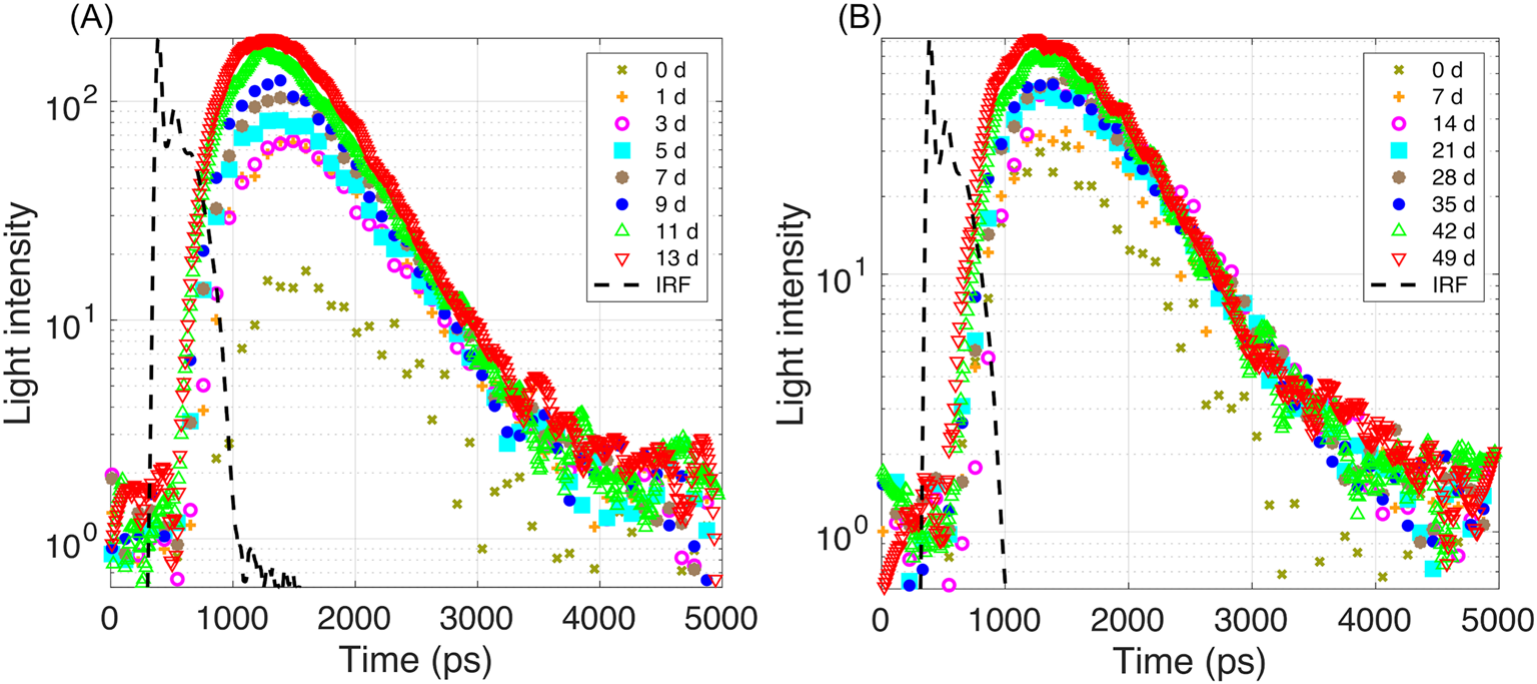
TRF at wavelength 846 nm collected from the kiwifruit during (**A**) shelf-life and in (**B**) cold storage conditions, respectively.

Figure 7A shows the TRP at the 846 nm wavelength of a representative sample under the shelf-life condition. It illustrates that longer storage time contributed to increased transmitted light intensity and decreased transmission time. The full width at half maximum of the IRF was 159.83 ps. It should be noted that light illumination was adjusted to be stronger than that used for the measurements of sliced samples. Figure 7B shows the TRP collected from another kiwifruit that under the cold storage condition, with the same tendency seen under the shelf-life condition, longer storage time contributed to an increase in transmitted light intensity and a decrease in transmission time. However, the speed of such changes was slower than that under the shelf-life condition. This agrees with the tendency of measured firmness values (Fig. 6), suggesting the importance of accessing the ripening process under different storage conditions.

**Figure 8:**
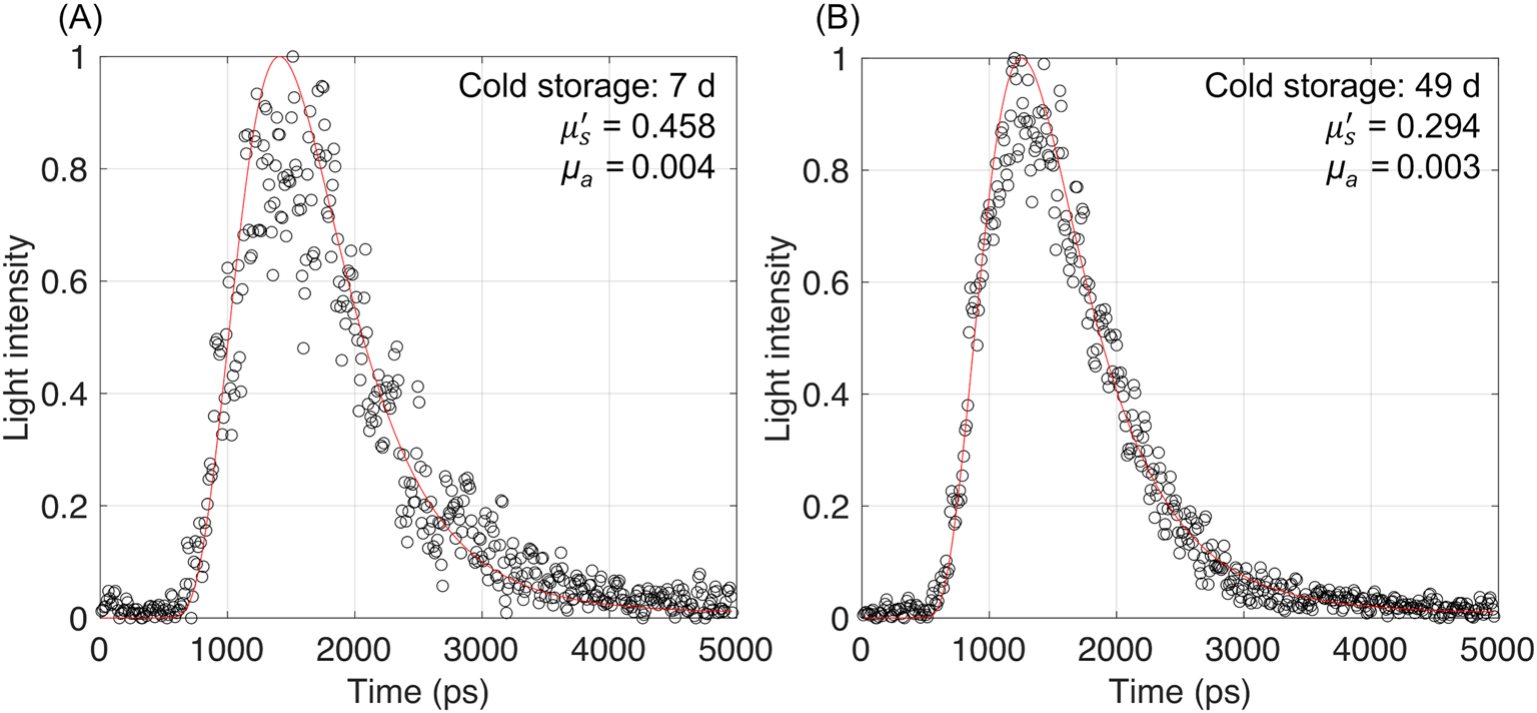
Estimated *μ*_a_ and ${\mu }_{s}^{\prime}$ values by fitting the TRP of the same kiwifruit during cold storage for (**A**) 7 and (**B**) 49 days, respectively.

Figure 8 shows one example of separating the light scattering effects from that of light absorption by fitting the TRP (black empty circles), which is convolved with the IRF, and the analytical solution of the diffusion approximation of the transport equation (red solid line). Tables 1 and 2 summarize the estimated *μ*_a_ and ${\mu }_{s}^{\prime}$ values of every ten kiwifruit during shelf-life and cold storage, respectively. The fluctuations in the range of ${\mu }_{s}^{\prime}$ among the ten samples could be mainly caused by the initial quality variation. Overall, the experimental results demonstrate that the ${\mu }_{s}^{\prime}$ inside kiwifruit tends to decrease during postharvest storage and is more evident under shelf-life than in cold storage conditions.

**Table 1:** Estimated *μ*_a_ and ${\mu }_{s}^{\prime}$ values of the ten kiwifruit during shelf-life storage.

Sample number	*μ*_a_ (mm^−1^)								${\mu }_{s}^{\prime}$ (mm^−1^)							
	0d	1d	3d	5d	7d	9d	11d	13d	0	1d	3d	5d	7d	9d	11d	13d
1	0.015	0.021	0.011	0.011	0.010	0.012	0.018	0.007	1.011	0.965	0.796	0.512	0.471	0.709	0.713	0.426
2	0.008	0.007	0.006	0.004	0.005	0.005	0.006	0.006	0.730	0.340	0.333	0.243	0.289	0.358	0.292	0.250
3	0.008	0.009	0.011	0.008	0.007	0.010	0.008	0.008	0.664	0.668	0.534	0.456	0.430	0.503	0.435	0.456
4	0.009	0.011	0.010	0.006	0.011	0.011	0.010	0.011	0.753	0.959	0.584	0.426	0.712	0.552	0.553	0.547
5	0.011	0.007	0.008	0.007	0.005	0.008	0.008	0.005	0.890	0.506	0.475	0.436	0.532	0.391	0.349	0.386
6	0.008	0.014	0.015	0.011	0.008	0.007	0.007	0.008	0.638	0.704	0.662	0.529	0.484	0.411	0.450	0.583
7	0.013	0.011	0.008	0.007	0.009	0.008	0.007	0.008	0.831	0.567	0.457	0.483	0.482	0.437	0.374	0.395
8	0.011	0.010	0.005	0.008	0.005	0.013	0.011	0.012	0.757	0.629	0.575	0.681	0.421	0.583	0.689	0.620
9	0.011	0.007	0.010	0.007	0.010	0.007	0.006	0.008	0.707	0.471	0.532	0.394	0.441	0.380	0.437	0.336
10	0.011	0.012	0.010	0.009	0.011	0.009	0.008	0.009	0.760	0.621	0.661	0.510	0.596	0.497	0.454	0.508

**Table 2:** Estimated *μ*_a_ and ${\mu }_{s}^{\prime}$ values of the ten kiwifruit during cold storage.

Sample number	*μ*_a_ (mm^−1^)								${\mu }_{s}^{\prime}$ (mm^−1^)							
	0d	7d	14d	21d	28d	35d	42d	49d	0d	7d	14d	21d	28d	35d	42d	49d
1	0.008	0.004	0.008	0.005	0.011	0.003	0.005	0.003	0.573	0.458	0.549	0.335	0.440	0.331	0.324	0.294
2	0.006	0.010	0.011	0.007	0.006	0.006	0.010	0.011	0.523	0.557	0.735	0.419	0.501	0.409	0.448	0.374
3	0.010	0.041	0.013	0.010	0.012	0.018	0.008	0.008	0.505	1.800	0.742	0.612	0.784	1.000	0.654	0.594
4	0.018	0.014	0.017	0.010	0.014	0.009	0.010	0.008	1.000	0.704	0.706	0.497	0.560	0.358	0.341	0.333
5	0.009	0.014	0.013	0.011	0.012	0.020	0.011	0.007	0.553	1.154	1.058	0.738	0.974	1.145	0.860	0.676
6	0.009	0.016	0.007	0.006	0.009	0.008	0.008	0.010	0.532	0.653	0.471	0.408	0.480	0.387	0.475	0.410
7	0.008	0.022	0.013	0.013	0.009	0.008	0.009	0.006	0.659	0.739	0.479	0.608	0.431	0.411	0.454	0.444
8	0.023	0.007	0.010	0.004	0.006	0.006	0.006	0.006	1.146	0.372	0.537	0.297	0.307	0.260	0.297	0.257
9	0.007	0.006	0.007	0.004	0.006	0.005	0.004	0.004	0.481	0.380	0.473	0.378	0.333	0.368	0.286	0.255
10	0.013	0.007	0.011	0.008	0.007	0.004	0.011	0.006	0.809	0.460	0.740	0.500	0.520	0.460	0.690	0.420

Tables 3 and 4 also summarize the *r* values between the estimated values of *μ*_a_ and ${\mu }_{s}^{\prime}$ and the storage time of every ten kiwifruit under shelf-life and cold storage conditions, respectively. Overall, there was an extremely unstable correlation between the *μ*_a_ and the storage time, suggesting that the *μ*_a_ at 846 nm has little relation to the fruit softening process. In contrast, fruit storage time and the ${\mu }_{s}^{\prime}$ (846 nm) of the same fruit that was negatively correlated (the longer the time, the lower the ${\mu }_{s}^{\prime}$) for both storage conditions. Numerous research investigations have demonstrated a correlation between the ${\mu }_{s}^{\prime}$ properties of fruit and textural attributes, including factors like firmness, cell size, and so on^31,51^. In the present study, the ${\mu }_{s}^{\prime}$ values and firmness of kiwifruit flesh exhibited a simultaneous reduction over the storage period. Comparable positive associations between ${\mu }_{s}^{\prime}$ and firmness were observed in 'Golden Delicious' and 'Granny Smith' apples, as indicated by Cen et al.^22^. However, Rowe et al. reported contrasting correlations for 'Royal Gala' apples with the ${\mu }_{s}^{\prime}$ range of 550–900 nm^52^, while Qin and Lu identified contrasting associations for 'Golden Delicious' apples at ${\mu }_{s}^{\prime}$ of 780 nm^53^, and Vanoli et al. found similar trends for ‘Braeburn’ apples at ${\upmu }_{\mathrm{s}}^{{{\prime}}}$ of 800 nm^15^. This could be due to the change of flesh texture from firm, crispy and juicy at harvest to mealy at the end of shelf life^54^. Since the ${\mu }_{s}^{\prime}$ was found to be predominantly influenced by both porosity and pore surface density, highlighting their significance as primary physical parameters for predicting scattering behavior^55,56^. In addition to the expected increase in light scattering caused by water loss, it is crucial to acknowledge that other factors can counterintuitively lead to a decrease in light scattering. These factors include the depolymerization of cell walls^48^, the loss of rigidity within pectic domains, and the presence of weakly bound water, which exhibits thermodynamically behavior similar to pure water^49^, could reduce the light scattering inside kiwifruit during post-harvest storage. Such phenomena agree with Burdon and Clark’s study^57^, which indicates that fruit water content decreases with decreased water potential. Slight moisture loss can cause subtle quality changes in color and texture, and critical moisture loss can cause severe damage to turgidity, firmness, flavor, and nutrition^58^. Furthermore, Taglienti et al. showed that free water becomes the prevailing fraction of the total aqueous protons in kiwifruit samples stored at 20 °C but does not significantly alter during 0 °C storage, even after several weeks.

**Table 3:** Correlation between the measured parameters and storage time of every ten kiwifruit during shelf-life storage.

Sample number	L^*^	a^*^	b^*^	a^*/^b^*^	*Chroma*	*Hue*	${\mu }_{s}^{\prime}$	*μ*_a_	PC1 score
1	0.68	− 0.28	− 0.26	− 0.04	− 0.03	0.04	− 0.74	− 0.46	0.95
2	0.53	0.56	0.44	− 0.04	− 0.05	0.04	− 0.60	− 0.49	0.97
3	− 0.86	0.39	− 0.10	0.26	0.24	− 0.27	− 0.81	− 0.33	0.96
4	− 0.56	− 0.24	− 0.50	0.27	0.30	− 0.26	− 0.58	0.27	0.98
5	− 0.50	0.11	− 0.04	0.26	0.26	− 0.26	− 0.72	− 0.61	0.98
6	− 0.16	0.70	0.58	0.24	0.21	− 0.24	− 0.68	− 0.62	0.74
7	− 0.41	− 0.28	− 0.36	0.11	0.11	− 0.10	− 0.78	− 0.68	0.98
8	0.57	− 0.46	− 0.34	− 0.36	− 0.34	0.37	− 0.23	0.33	0.96
9	0.51	0.15	0.50	− 0.69	− 0.69	0.68	− 0.77	− 0.47	0.98
10	0.69	− 0.38	− 0.12	− 0.19	− 0.21	0.18	− 0.84	− 0.76	0.98

**Table 4:** Correlation between the measured parameters and storage time of every ten kiwifruit during cold storage.

Sample number	L*	a*	b*	a*^/^b*	*Chroma*	*Hue*	${\mu }_{s}^{\prime}$	*μ*_a_	PC1 score
1	0.47	− 0.11	0.21	− 0.44	− 0.46	0.44	− 0.86	− 0.41	0.97
2	− 0.48	− 0.03	− 0.46	0.54	0.55	− 0.54	− 0.63	0.2	0.88
3	− 0.34	0.41	− 0.11	0.59	0.59	− 0.59	− 0.29	− 0.43	0.82
4	0.49	0.06	0.16	− 0.12	− 0.12	0.12	− 0.53	− 0.48	0.64
5	0.19	− 0.26	− 0.52	0.38	0.38	− 0.39	− 0.83	− 0.43	0.89
6	− 0.34	0.01	− 0.44	0.53	0.52	− 0.54	− 0.65	− 0.23	0.60
7	0.57	0.20	0.46	− 0.21	− 0.21	0.21	− 0.78	− 0.56	0.94
8	− 0.25	0.03	0.03	− 0.02	− 0.01	0.02	− 0.72	− 0.65	0.92
9	− 0.68	− 0.47	− 0.66	− 0.04	− 0.03	0.04	− 0.88	− 0.86	0.87
10	0.38	0.00	0.03	0.00	− 0.01	0.00	− 0.48	− 0.49	0.91

**Figure 9:**
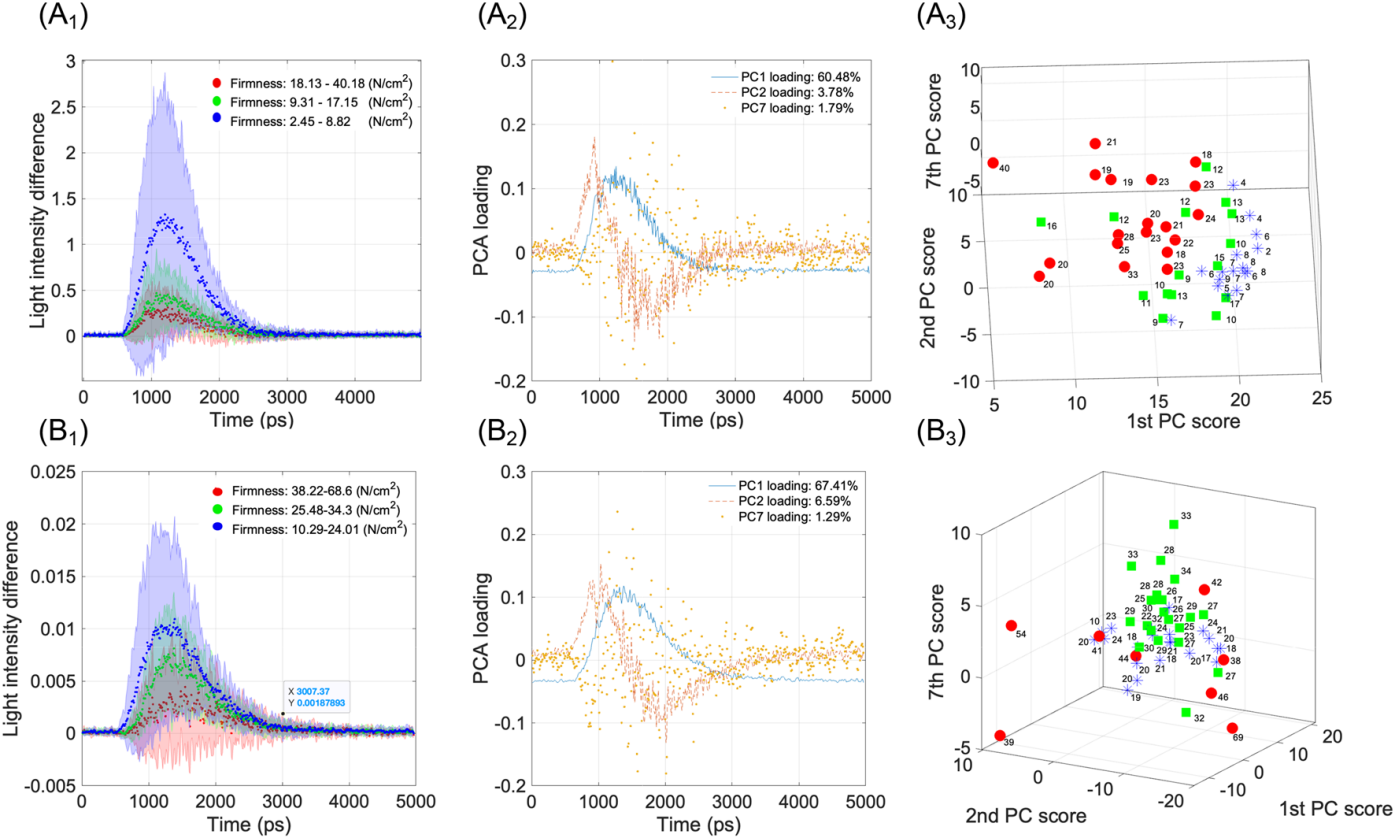
TRP difference spectral data (**A**_**1**_ shelf-life and **B**_**1**_ cold storage), with their PC1, PC2, and PC7 loadings (**A**_**2**_,**B**_**2**_), and the three PC scores of the TRP difference spectra (**A**_**3**_,**B**_**3**_). The number in each scatter plot show the firmness reference values.

Since the *r* values of the ${\mu }_{s}^{\prime}$ were lower than those of the PC 1 scores, suggesting the TRTS measurement of the intact kiwifruit, the curve-fitting method should be further improved. Nevertheless, this experiment indicated that the use of ${\mu }_{s}^{\prime}$ at 846 nm was more robust than the use of color parameters or *μ*_a_ at the same wavelength to monitor the kiwifruit softening process during postharvest storage. To further develop a nondestructive fruit softening progress prediction method based on the light scattering phenomena, the TRP difference spectra were first classified into three classes based on their measured firmness values (Fig. 9A_1_: shelf-life and B_1_: cold storage). It is evident that the softer samples tend to have higher light intensity with faster transmitted time, i.e., lower ${\mu }_{s}^{\prime}$. Figure 9A_2_,B_2_ show their PC1, PC2, and PC7 loadings. The accumulated contribution rate of the three PC scores was approximately 66.05% and 75.29%, respectively. The scatter plots of the three PC scores are shown in Fig. 9A_3_,B_3_. The three PC scores exhibited significant variations that played a crucial role in effectively classifying the stages of kiwifruit softening, namely early, mid-, and late stages.

**Figure 10:**
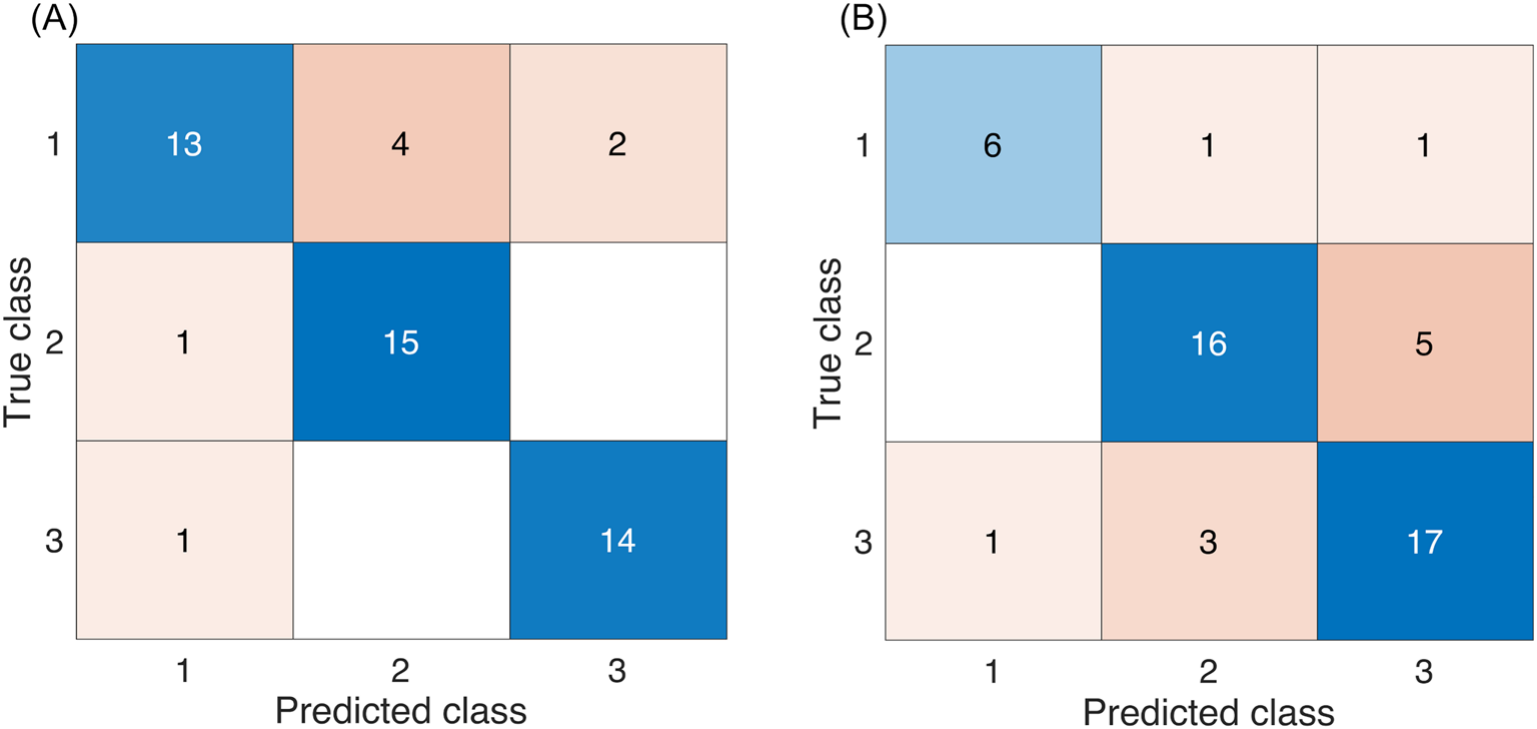
Prediction results for the softening stage of kiwifruit under (**A**) shelf-life and (**B**) cold storage conditions, respectively. In each matrix, each row corresponds to the predicted class, while each column represents the actual class (class 1 indicates the samples with the highest firmness values, followed by class 2 and class 3).

The classification results of the three softening classes of kiwifruit using the SVM and employing a five-fold cross-validation approach are presented in Fig. 10. The PC scores were utilized as representative features for training the SVM classification model based on the TRP spectra. Notably, classification accuracies of 84% and 78% were achieved for the shelf-life and cold storage conditions, respectively. It demonstrated the usefulness of utilizing the light scattering information for monitoring the kiwifruit softening stages.

## Conclusion

This study developed a TRTS measurement system that mainly includes a picosecond pulsed laser with a wavelength of 846 nm and a streak camera. The light scattering characteristics of the kiwifruit samples during the softening (i.e., their mechanical properties) were compared. Overall, the ${\mu }_{s}^{\prime}$ exhibited a decreasing trend over time for both the shelf-life and cold postharvest storage conditions, with a more pronounced effect observed under the former condition. Furthermore, a successful mathematical model was developed to effectively classify the softening stages of early, mid-, and late using a single wavelength of 846 nm.

The experimental findings are anticipated to have significant implications in two key aspects. Firstly, they contribute to enhancing our comprehension of the intricate relationship between the spectral data obtained from the collected samples and their corresponding mechanical properties. Secondly, these results hold potential for driving advancements in the field of portable measurement systems, paving the way for the development of robust firmness prediction models that can operate independently of conventional multivariate statistical analysis methods. This progress is crucial for promoting efficient and reliable assessments of fruit firmness in various practical applications. Further experimental investigations are necessary to explore the variations in light scattering through the utilization of diverse data collection methods, encompassing a wide range of fruit types and postharvest storage conditions.

## Data Availability

The datasets used and/or analyzed during the current study available from the corresponding author on reasonable request.
